# Induction cisplatin–irinotecan followed by concurrent cisplatin–irinotecan and radiotherapy without surgery in oesophageal cancer: multicenter phase II FFCD trial

**DOI:** 10.1038/sj.bjc.6603328

**Published:** 2006-09-12

**Authors:** P Michel, A Adenis, F Di Fiore, E Boucher, M P Galais, L Dahan, X Mirabel, H Hamidou, J L Raoul, J H Jacob, M F Hellot, S Prod'Homme, B Paillot

**Affiliations:** 1 Unité d’oncologie digestive, Service d’Hepato-Gastroenterologie, CHU de Rouen, 1 rue de Germont 76031 Rouen Cedex, France; 2 Service d’Oncologie Medicale, Centre Oscar Lambret, 3 rue Frederic Combemale, BP307, 59020 Lille, France; 3 Département d’Oncologie Medicale, Centre Regional de Lutte Contre le Cancer, Eugene Marquis, 35062 Rennes Cedex, France; 4 Service d’Onclogie Digestive, Centre Regional de Lutte Contre le Cancer, Francois Baclesse, 3 avenue General Harris, 14076 Caen Cedex, France; 5 Unité d’Oncologie Digestive, Service d’Hepato-Gastroentérologie, CHU Timone, 264 rue Saint-Pierre 13365 Marseille Cedex 5, France; 6 Service de Radiotherapie, Centre Oscar Lambret, 3 rue Frederic Combemale, BP307, 59020 Lille, France; 7 Service de Radiotherapie Centre Régional de Lutte Contre le Cancer, Henri Becquerel, rue d’Amiens, 76000 Rouen, France; 8 Unité de Biostatistique, CHU de Rouen, 1 rue de Germont, 76031 Rouen Cedex, France; 9 Sabrina Prod’homme, Delegation à la Recherche Clinique, CHU de Rouen, 1 rue de Germont, 76031 Rouen Cedex, France

**Keywords:** oesophageal cancer, exclusive chemo-radiotherapy, induction chemotherapy

## Abstract

A recent phase I study showed that weekly cisplatin, irinotecan and concurrent radiotherapy can be administered with moderate toxicity in patients with oesophageal cancer. Patients with no prior treatment and oesophageal cancer stage I to III, performance status <3, caloric intake >1500 kcal day^−1^ were included. Chemotherapy, with cisplatin 30 mg m^−2^ and irinotecan 60 mg m^−2^, was administered at days 1, 8, 22, 29, and concurrently with radiotherapy at days 43, 50, 64 and 71. Radiotherapy was delivered with 50 or 50.4 Gy in 25 fractions/5 weeks. Forty-three patients were included, 10 stage I, 19 stage II and 14 stage III. Mean age was 59.2 years (range 44–79). A total of 30 out of 43 (69.8%) patients underwent all planned treatment. During induction chemotherapy, 14 severe toxicities of grade 3 or 4 in 10 patients (23.3%) were reported with 57.1% due to haematoxicity. During chemoradiotherapy, 31 severe toxicities of grade 3 or 4 with 64.5% due to haematotoxicity were reported in 18 patients. One toxic death occurred (diarrhoea grade 4). The complete clinical response rate was 58.1% (95% CI: 43.4–72.8%). Overall survival rate at 1 and 2 years was 62.8%, (95% CI, 58.3–77.3%) and 27.9% (95% CI, 13.4–41.3%), respectively. In conclusion, cisplatin–irinotecan–radiotherapy is an active and well-tolerated regimen feasible in out-patients.

Oesophageal cancer is an aggressive disease with a poor prognosis (Enzinger and Mayer, 2003). In the USA and Europe the number of new cases per year is 13 100 and 34 300, respectively (Jemal *et al*, 2002; Keighley, 2003). At the time of the diagnosis, more than 50% of patients have inoperable disease. In these patients, the more effective treatment in locally advanced disease is chemo-radiotherapy (Enzinger and Mayer, 2003). Moreover, in operable patients with locally advanced oesophageal cancer, the combination of chemotherapy and concurrent radiotherapy has been demonstrated to have an outcome similar to that of surgery after preoperative therapy (Bedenne *et al*, 2002; Stahl *et al*, 2005). The standard chemotherapy regimen associates 5 Fluorouracil (FU) with cisplatin (Herskovic *et al*, 1992; Cooper *et al*, 1999) and the recommended total dose of concurrent radiation is 50 Gy (Minsky *et al*, 2002). A number of drugs have been tested in oesophageal cancer (Enzinger *et al*, 1999; Geh, 2002). Recent trials combining weekly irinotecan with cisplatin have reported a response rate exceeding 50% (Ilson *et al*, 1999; Ajani *et al*, 2002). The active metabolite of irinotecan (SN38) increases the proportion of cells in the G2–M or M phase. These phases are the most radiosensitive of the cycle (Tamura *et al*, 1997). Phase I–II studies of weekly irinotecan and radiation therapy have been performed (Koukourakis *et al*, 1999; Takeda *et al*, 1999). Recently, phase I study of weekly with cisplatin–irinotecan chemotherapy followed by chemo-radiotherapy in locally advanced oesophageal cancer has been reported (Ilson *et al*, 2003). The dose-limiting toxicity was primarily myelosuppression, no grade 3 or 4 oesophagitis, diarrhoea or stomatitis was observed.

In the current multicentre phase II trial, our aim was to evaluate the efficacy and toxicity of cisplatin–irinotecan chemotherapy followed with chemo-radiotherapy as definitive treatment in patients with oesophageal cancer.

## PATIENTS AND METHODS

### Patient eligibility

All patients had histologically confirmed squamous cell carcinoma, adenocarcinoma or poorly differentiated non-small-cell carcinoma of the oesophagus. Participants were required to be at least 18 years of age and to provide written informed consent prior to treatment. Patients had an Eastern Cooperative Oncology Group (ecog) performance status ⩽2 (Karnofsky performance status of 70% or greater), caloric intake >1500 kcal d^−1^, serum albumin ⩾32 g l^−1^ and adequate haematological, renal, and hepatic function as defined by an absolute neutrophil count ⩾1.5 × 10^9^ l^−1^, platelets ⩾100 × 10^9^ l^−1^, serum creatinine ⩽120 *μ*mol l^−1^ and total serum bilirubin ⩽1.5 mg dl^−1^. Patients had no prior chemotherapy, radiotherapy or surgery. Patients with metastatic disease to supraclavicular or metastatic disease with biopsy-proven tumour invasion of the tracheobronchial tree or with tracheoesophageal fistula were not included. Patients with severe comorbid conditions, including cardiac disease graded as New York Heart Association class 3 or 4, or myocardial infarction within the previous 6 months were also excluded. Also patients with a prior history of malignancy, other than basal cell carcinoma of the skin, *in situ* cervical carcinoma, or head and neck carcinoma with complete response since 3 years of inclusion of the study were also not included. Patients with known Gilbert's syndrome were not included.

### Pretreatment evaluation and evaluation on study

Pretreatment evaluation included a detailed medical history and physical examination, a complete blood count, biochemical screening profile including liver function assessment and electrolytes, a prothrombin time and ECG. Radiologic evaluation included a CT scan of the chest and abdomen. Patients were required to undergo endoscopy with biopsy of the primary tumour. Endoscopy with ultrasonography (EUS) was optional. Bronchoscopy was performed in patients with tumours of the cervical or proximal thoracic oesophagus and in patients with squamous cell carcinoma. The 1983 AJCC staging system (Table 1) was used in this study according to published recommendations (Coia *et al*, 2000). Patients were examined the morning prior to each chemotherapy infusion during induction chemotherapy and combined chemo-radiotherapy. At 10–12 weeks after completion of therapy, an upper endoscopy with biopsy, and a CT scan were repeated to assess response. Clinical complete response was defined as no tumour detectable on oesophagus endoscopy and no appearance of lymph nodes or distant metastasis on CT scan (Kelsen *et al*, 1983; Jones *et al*, 1999). In all other cases, the patients were considered in the absence of response. Surgery was not mandated on protocol. When patients achieved a complete clinical response, immediate follow-up was carriedout at the discretion of the investigator. Upper endoscopy and CT scan of the chest and abdomen were performed annually. Dysphagia was evaluated prior to therapy and after completion of induction chemotherapy using a previously published dysphagia scale (Ogilvie *et al*, 1982). All toxicity was graded using the National Cancer Institute Common Toxicity Criteria version 2.0.

### Treatment plan

**Figure 1:**
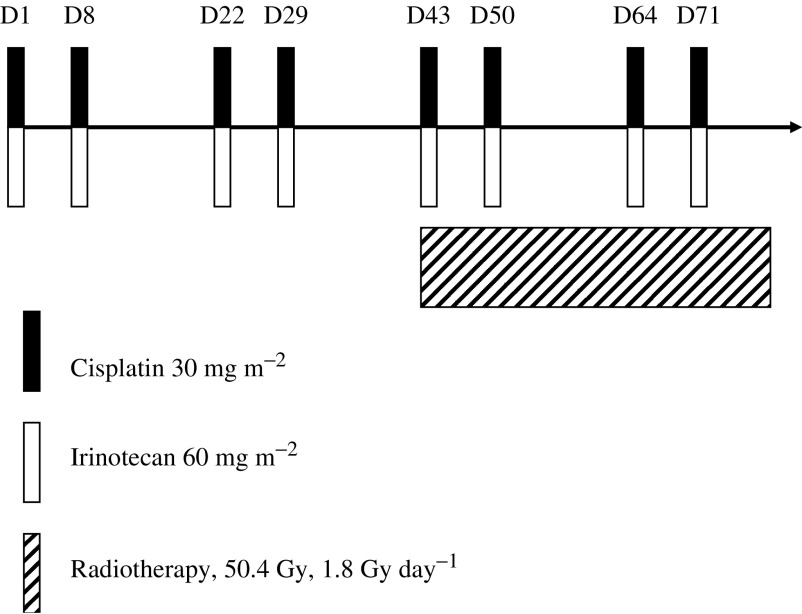
Treatment flow chart.

The treatment procedure is outlined in Figure 1. Therapy was delivered in two phases: four induction chemotherapy courses were administered on days 1, 8, 22, 29 with the third and sixth week used as rest weeks. Four chemotherapy courses concurrent with radiotherapy (courses 5 to 8) were administered days 43, 50, 64 and 71 with the ninth week used as a rest week. Antiemetic therapy, with steroid and 5-HT3 receptor antagonist was recommended. Hydration with 500 ml of intravenous fluid was performed before cisplatin at a dose of 30 mg m^−2^ as a 30-min infusion. After cisplatin, irinotecan was administered at a dose of 60 mg m^−2^ as a 30-min infusion. As required, atropine 0.5 mg was given to patients who developed abdominal cramps or diarrhoea within 1 h of irinotecan infusion. Written information regarding the treatment of diarrhoea as delayed toxicity was routinely given at the first chemotherapy. All diarrhoeas were treated by loperamide. To continue induction chemotherapy, patients were required to maintain a WBC ⩾3.0 × 10^9^ l^−1^, absolute neutrophil count ⩾1.5 × 10^9^ l^−1^, platelet count ⩾100 × 10^9^ l^−1^, serum creatinine ⩽120 *μ*g l^−1^ and diarrhoea toxicity ⩽grade 2. No prophylactic use of granulocyte colony-stimulating factor was planned.

### Radiation therapy

Radiation therapy was delivered with megavoltage equipment using a multiple-field technique. Patients were treated 5 days per week at 1.8 or 2 Gy day^−1^ to a total dose of 50.4 or 50.0 Gy. All fields were treated each day. Treatment was delivered to three or four fields (anterior–posterior/posterior–anterior and opposed laterals) in order that the dose did not vary by more than 5% over the entire target volume. The prescribed total dose was prescribed at the reference point (isocentre) of the PTV, which covered the volume at risk. The upper and lower borders of the radiation field were 3–5 cm beyond the primary tumour. The lateral, anterior and posterior borders of the field were ⩾2 cm beyond the borders of the primary tumour. The tumour size was defined by CT scan. The primary and regional lymph nodes were also included.

### Statistical design

The primary end point of the study was clinical complete response rate. The study was designed using a two-stage design (Bellisant *et al*, 1996). Assuming clinical complete response rate at 60% with alpha risk 5% and power 80%, the number of patients necessary was calculated at 43. In the first stage, 34 patients were accrued. If clinical complete response was observed in less than 17 patients, the trial was considered closed due to ineffectiveness of studied regimen. If more than 17 patients presented a complete clinical response, inclusion was continued to 43 patients. Analysis was performed in intent to treat. Quantitative data were presented as mean or median. Qualitative data were presented with frequency and percentage. The proportion of patients with clinical complete response was used to estimate the true response rate with a 95% confidence interval (95% CI). Overall survival and survival without recurrence rate at 1 and 2 years was estimated. The dysphagia scale used had five stages (Ogilvie *et al*, 1982). For results analysis patients were pooled in two groups (1–2, >2). Dysphagia prior to and after induction chemotherapy was compared. Then, relationship between dysphagia after induction chemotherapy and response was evaluated. These groups were compared using the Wilcoxon–Mann–Whitney test.

## RESULTS

Between February 2003 and December 2003, 43 patients were included. Patient characteristics are listed in Table 2. Histological type was squamous cell carcinoma in 38 out of 43 patients (88.4%). At the inclusion, 12 out of 43 patients (27.9%) presented a severe weight loss (>15%) and 26 out of 43 patients (60.5%), a moderate deterioration of performance status (ECOG 1).

### Treatment

Of the 43 patients included, 30 (69.8%) completed treatment as planned, 35 patients (81.4%) completed the four cycles of induction chemotherapy and 34 patients (79.1%) completed the total doses of radiation therapy. Radiotherapy with 2 Gy per fraction was performed in 31 patients (79.5%). In eight patients, a radiotherapy protocol violation recorded with fractions number superior to 25. The total dose was 56 Gy in 1, 60 Gy in 4, 62 Gy in 1 and 64 Gy in 1. In eight patients (18.6%) treatment was stopped due to toxicity (4 haematological, 4 nonhaematological). In four patients, treatment was stopped after cycle 7 due to nonadherence to protocol without toxic effect reported. One patient declined to undergo the last cycle of induction chemotherapy. During induction chemotherapy period, dose attenuation was required in less than 10% of patients: course 3 (cisplatin 1 patient, irinotecan 4 patients); course 4 (irinotecan 2 patients). Treatment delay occurred in 15 and 5.7% of patients, respectively, at course 3 and 4. The delay was due to haematological toxicity in 87.5% of cases. During chemo-radiotherapy period, dose attenuation was performed at courses 5, 6, 7 and 8, in 22.1, 23.0, 25.7 and 24.3% of patients for cisplatin and in 22.1, 28.6, 28.6 and 30.3% of patients for irinotecan, respectively. Treatment delay occurred in 21.0, 20.0, 20.0 and 15.6% of patients, respectively, at courses 5, 6, 7 and 8. Treatment delay was due to haematological toxicity in 24 out of 27 (88.9%) of cases, the other causes were dysphagia (2 patients) and deep thrombosis (one patient). The most common cause of dose attenuation was haematologic toxicity.

### Toxicity

Toxicities observed during induction chemotherapy are listed in Table 3. During this period, 246 toxicities were reported with 56.9% due to haematoxcicity. Fourteen severe toxicities of grade 3 or 4 in 10 patients (23.3%) were reported with 57.1% due to haematoxcicity. Hospitalisation was required in five patients during this period, due to deficient nutritional intake (two patients), myocardial infarction (one patient), severe diarrhoea (grade 4) (one patient) and tracheoesophageal fistula (one patient).

Toxicities observed during chemoradiotherapy are listed in Table 4. During this period, 311 toxicities were reported with 72.8% due to haematotoxicity. Thirty-one severe toxicities of grade 3 or 4 with 64.5% due to haematotoxicity were reported in 18 patients. During this period, no grade 3–4 alopecia was recorded. Only one deep thrombosis was observed. The other non-haematological toxicities were tachycardia (one), and common toxicities with chemotherapy (functional kidney failure: one, mucositis: one) and radiotherapy (dysphagia: two, asthenia: four). Seventeen hospitalisations in 13 patients were required. Hospitalisations were required for vomiting, dysphagia or oesophagitis (six), only biological disorders (seven), sepsis (one), asthenia (one) and deep thrombosis (one). One treatment-related death occurred: 4 days after the cycle 7, patient 31 presented with a severe diarrhoea grade 4 with vomiting. This patient was hospitalised in the emergency ward. He developed aplasia and kidney failure, and later died from septic shock.

Severe adverse events were reported in 24 patients (55.8%) during the treatment; 11 (25.6%) and 13 (30.2%) of patients during induction chemotherapy and chemo-radiotherapy, respectively.

### Response and survival

Twenty-five of 43 included patients (58.1%; 95% CI, 43.4–72.8%) achieved a complete clinical response. In 14 patients, the complete clinical response was defined by endoscopy with biopsy, in other patients only macroscopic characteristics were used. Thirteen patients (30.2%; 95% CI, 16.5–43.9%) were in the absence of response as previously defined (i.e. partial response, stable disease or progression). Five patients were not available for response, as four patients discontinued treatment prior to radiotherapy and one patient died before evaluation. One patient was lost to follow-up after the first cycle of chemotherapy, the 42 other patients were followed-up at 2 years or to death. Overall survival rate at 1 and 2 years were 62.8% (95% CI, 58.3–77.3%) and 27.9% (95% CI, 13.4–41.3%), respectively. The disease free survival rate at 1 and 2 years was 23.2% (95% CI, 10.6–35.8%) and 16.7% (95% CI, 5.6–27.8%). Three patients underwent surgery during follow-up period for suspected local recurrence. In one case (i.e. poor differentiated tumour) oesophageal EUS performed 2 months after the treatment suggested a stable disease (uT3) when endoscopy was normal. The pathologic examination of the surgical specimen showed the absence of cancer cells and confirmed the clinical complete response. In the two other cases, local recurrence was confirmed by biopsies, in one patient the tumour was not resectable.

### Dysphagia

Only 12 patients (29.3%) had dysphagia (grade>2) prior to the treatment. After induction chemotherapy and chemo-radiotherapy, 15 (38.5%) and 6 (17.1%) patients, respectively, experienced dysphagia (grade>2). The differences were not statistically significant with *P*=0.565 and 0.099, respectively (Table 5). No significant correlation was found between dysphagia after induction chemotherapy and clinical response after the treatment (*P*=0.797). The variation of weight during the treatment regimen was mean −0.3 kg (range: +2 to −13 kg).

## DISCUSSION

The present study is to our knowledge the first phase II trial evaluating in a population of inoperable patients with the regimen defined in phase I (Ilson *et al*, 2003). The main results of the present study were the complete clinical response rate of 58.1% (95% CI, 43.4–72.8%), overall survival rate of 27.9% (95% CI: 13.4–41.3%) at 2 years and the absence of significant improvement of dysphagia after induction chemotherapy. Compliance was satisfactory in 69.8% patients who completed treatment as planned although a severe adverse event was reported in 24 patients (55.8%).

The combination of weekly cisplatin–irinotecan in metastatic oesophageal cancer showed an objective response rate of 57% (Ilson *et al*, 1999). In the present study, the clinical complete response rate obtained with cisplatin–irinotecan and radiotherapy regimen was 58.1% (CI 95%: 43.4–72.8%), in a range similar to that reported with a FU–cisplatin and radiotherapy regimen (Seitz *et al*, 1990; Poplin *et al*, 1996; Geh, 2002). The overall survival rate at 2 years of 27.9% (CI 95%: 13.4–41.3%) in our study was similar to the result of standard treatment of definitive concomitant reported in the RTOG-8501 trial and the recent phase III study with chemo-radiotherapy as definitive treatment (Herskovic *et al*, 1992; Bedenne *et al*, 2002; Minsky *et al*, 2002; Stahl *et al*, 2005). All results published with cisplatin–irinotecan regimen suggest that its efficacy is similar to that of a standard regimen with FU–cisplatin for response rate and overall survival (Geh, 2002).

In the present study, no significant improvement of dysphagia was obtained by induction chemotherapy. However, in other studies using this combination of irinotecan–cisplatin in chemotherapy, a significant improvement of dysphagia was reported (Ilson *et al*, 1999; Tew *et al*, 2005). Ilson *et al* observed dysphagia resolution in 70% of patients after four chemotherapy courses. In our study, only 28.1% of patients reported dysphagia resolution after induction chemotherapy.

The development of regimens with induction chemotherapy has been significant; therefore, this regimen should have a similar efficacy as compared to the standard regimen. In contrast, the optimisation of the cisplatin–irinotecan–radiotherapy regimen could be potentially feasible. Epidermal growth factor receptor (EGFr) antibody or inhibitor could also increase the efficacy of the regimen: first by improving the therapeutic effect of irinotecan via EGFr antibodies as demonstrated *in vitro* (Prewett *et al*, 2002), second by the specific approach as demonstrated in head and neck cancer, by using radiation therapy and EGF receptor antibodies (Bonner *et al*, 2006; Pfister *et al*, 2006). The association cisplatin–irinotecan–radiotherapy with EGFr antibody warrants further study.

In the present study, planned treatment was performed in 69.8% of patients. This compliance is similar to 68% observed with 5FU and cisplatin in the RTOG 85-01 trial (Herskovic *et al*, 1992; Cooper *et al*, 1999). A severe adverse event was observed in 44 and 55.8% of patients in the RTOG 85-01 study and our phase II, respectively. In the present study, two patients (4.6%) presented life-threatening events compared with 20% in RTOG study. Haematotoxicity represented 62.2% of severe adverse events as in other regimens evaluated in patients with oesophageal cancer (Geh, 2002). In RTOG 85-01 trial, grade 3–4 esophagitis occurred in 33% of patients receiving chemo-radiotherapy (Herskovic *et al*, 1992). In present study only three patients (7%) reported a grade 3–4 oesophageal toxicity. The incidence of clinical deep thrombosis was poor with only one case reported in our study.

In conclusion, cisplatin–irinotecan–radiotherapy regimen could be considered effective and its tolerance is similar to the standard treatment. Moreover, the regimen can be performed on an outpatient basis. Furthermore, chemotherapy induction and the possibility to optimise the association with EGFr antibody warrants further studies with the combination irinotecan–cisplatin and radiotherapy in patients with oesophageal cancer.

## Figures and Tables

**Table 1:** The 1983 AJCC staging system for oesophageal cancer

**Stage**	**Criterion**
I	<5 cm in length, nonobstructing, noncircumferential
II	> 5 cm in length or obstructing or circumferential
III	Evidence of extra oesophageal spread (computed tomography scan indicating invasion of surrounding structures or clinical evidence such, as recurrent laryngeal nerve involvement, positive pleural effusion, tracheoesophageal fistula, sympathetic nerve involvement, phrenic nerve involvement or widened mediastinum on chest radiograph)

**Table 2:** Patient characteristics

*N*=43	
Age in years (mean)	59.2 yrs (60.9 yrs)
Sex ratio	30 males/4 females
	
*Histology n (%)*	
Squamous cell carcinoma	38 (88.4%)
Adenocarcinoma	4 (9.3%)
Poor differenced	1 (2.3%)
	
*Tumour n (%)*	
Proximal	15 (34.9%)
Middle	19 (44.2%)
Distal	9 (20.9%)
	
*Dysphagia (Atkinson score): n (%)*	
Grade>2	12 (29.3%)
	
*Performance status ECOG: n (%)*	
0	17 (39.5%)
1–2	26 (60.5%)
	
*Weight loss n (%)*	
<15%	31 (72.1%)
>15%	12 (27.9%)
	
*Tumour stage, UICC 1983: n (%)*	
Stage I	10 (23.2%)
Stage II	19 (44.2%)
Stage III	14 (32.6%)
Serum albumin (mean in g l^−1^)	39.5 g l^−1^
Tumour diameter (CT scan, median in mm)	34.5 mm
	
*Endoscopic Ultrasonography (n=12)*	
T3	9 (75%)
N+	6 (50%)
M1a (celiac node)	1 (8.3%)

**Table 3:** Toxicity episodes: induction chemotherapy

	**Grade 1**	**Grade 2**	**Grade 3**	**Grade 4**
Alopecia	9	9	0	0
Anaemia	75	13	0	0
Diarrhoea	26	3	1	1
Fever	1	1	0	0
Infection+neutropenia	0	1	0	0
Nausea-vomiting	32	13	1	0
Neutropenia	22	11	6	1
Febrile neutropenia	0	4	0	0
Mucositis	6	0	0	0
Thrombopenia	5	1	1	0
Deep thrombosis	0	0	0	0
Heart	0	0	0	1
Fatigue	0	0	0	1
Esotracheal fistula	—	—	—	1

**Table 4:** Toxicity episodes: combined chemoradiotherapy

	**Grade 1**	**Grade 2**	**Grade 3**	**Grade 4**
Alopecia	13	7	0	0
Anaemia	97	36	5	0
Diarrhoea	11	3	0	0
Fever	3	0	0	0
				
Oesophagitis^a^	21	17	5	1
Infection+neutropenia	0	0	0	1
Nausea-vomiting	27	0	0	0
Leuconeutropenia	31	11	9	3
Neutropenia and fever	1	16	0	1
Mucositis	9	0	1	0
Thrombopenia	13	2	2	0
Deep thrombosis	0	0	1	0
Heart	0	0	1	0
Sepsis	0	0	0	1
Kidney	0	0	1	0
Asthenia	0	0	4	0

a Oesophagitis defined by painful dysphagia and/or thoracic burning.

**Table 5:** Dysphagia

**Atkinson score**	**Before treatment *N*=41**	**End of induction chemotherapy *N*=39**	**End of chemoradiotherapy *N*=35**
1	4	11	16
2	25	13	13
3	8	8	2
4	4	6	3
5	0	1	1
